# Climate Factors Influencing Coccidioidomycosis Seasonality and Outbreaks

**DOI:** 10.1289/ehp.7786

**Published:** 2005-03-03

**Authors:** Andrew C. Comrie

**Affiliations:** Department of Geography and Regional Development, University of Arizona, Tucson, Arizona, USA

**Keywords:** climate, *Coccidioides*, coccidioidomycosis, environment, meteorologic factors, rain, seasonal variation, southwestern United States, weather

## Abstract

Although broad links between climatic factors and coccidioidomycosis have been established, the identification of simple and robust relationships linking climatic controls to seasonal timing and outbreaks of the disease has remained elusive. Using an adaptive data-oriented method for estimating date of exposure, in this article I analyze hypotheses linking climate and dust to fungal growth and dispersion, and evaluate their respective roles for Pima County, Arizona. Results confirm a strong bimodal disease seasonality that was suspected but not previously seen in reported data. Dispersion-related conditions are important predictors of coccidioidomycosis incidence during fall, winter, and the arid foresummer. However, precipitation during the normally arid foresummer 1.5–2 years before the season of exposure is the dominant predictor of the disease in all seasons, accounting for half of the overall variance. Cross-validated models combining antecedent and concurrent conditions explain 80% of the variance in coccidioidomycosis incidence.

Coccidioidomycosis, or valley fever, is caused by inhalation of spores from *Coccidioides immitis* and *Coccidioides posadasii*. These dimorphic soil fungi are endemic to the deserts of the southwestern United States, Mexico, and elsewhere in Central and South America (Fisher et al. 2002; Kolivras et al. 2001). Although approximately 60% of people infected with the disease are asymptomatic, others experience mild influenza-like symptoms, and a small percentage experience severe effects and sometimes death resulting from dissemination of the disease to other parts of the body (Kolivras et al. 2001). Those at greatest risk for coccidioidomycosis infection include immunocompromised patients, young children, the elderly, and members of several ethnic minorities in the United States (Kolivras et al. 2001; Pappagianis 1988). In Arizona alone, > 2,000 cases per year have been reported (Komatsu et al. 2003), and the incidence of coccidioidomycosis is greater than that for other emerging infectious diseases in the region such as West Nile virus [Centers for Disease Control and Prevention (CDC) 2004a]. The number of Arizona cases is likely to exceed 3,000 by the end of 2004 (CDC 2004b).

Environmental conditions appear to have an important impact on coccidioidomycosis incidence. The current Arizona coccidioidomycosis epidemic has been linked to climate conditions (Kolivras and Comrie 2003; Komatsu et al. 2003; Park et al. 2005), whereas California experienced an epidemic in the 1990s that was possibly linked to drought conditions (Jinadu 1995). Initial links between climate conditions and the disease were identified several decades ago (Hugenholtz 1957; Maddy 1965). It is only recently that further details on climate and coccidioidomycosis have been published (Kolivras and Comrie 2003; Komatsu et al. 2003). These studies identified associations linking climate and other factors to seasonal patterns of coccidioidomycosis and to interannual variability and trends in the disease. Significant variables included drought indices, lagged precipitation, temperature, wind speed, and dust during the preceding 1 or more years. The relationships to coccidioidomycosis were quite complex, however, perhaps because of disease data issues outlined below. In this article I aim to identify simple and robust relationships linking climatic controls to seasonal timing and outbreaks of the disease, which until now have remained elusive and poorly understood. Important public health opportunities exist if environmental factors controlling coccidioidomycosis outbreaks and trends can be better comprehended, including the timing and degree of mitigation efforts such as soil treatment and the development of an advance warning system for public health management.

Part of the reason for the current state of knowledge has been the lack of high-quality disease data series. In fact, a major challenge to understanding more about the links between climate and infectious disease continues to be the difficulty in obtaining regular time series of disease data (National Research Council 2001). This is especially true for coccidioidomycosis with respect to data on *Coccidioides* in the soil or atmosphere. The current environmental detection method using laboratory mice is expensive and time-consuming, and although there is ongoing research into more rapid detection techniques (e.g., using polymerase chain reaction analysis to detect the fungus in soil samples), it will be several years before time series of such data become available. In the absence of suitable data on the environmental variability of the fungus itself, there is a need to exploit epidemiologic data in different ways to better identify the role of environmental controlling factors such as climate. Thus, for now, disease incidence data offer the best (and only) available multiyear time series for comparison with climatic conditions.

The use of human disease data to study potential relationships to climate conditions introduces numerous methodologic and analytical issues related to collection and reporting. Incidence data do not provide a homogeneous time series because of changes in reporting requirements, changes in population demographics, and the introduction of new diagnostic tests. In addition, the reported data necessarily contain imprecise estimations of disease onset dates because of various factors including patient recall, incorrect or delayed diagnoses caused by displacement of diagnoses during the respiratory disease season, and the variability in disease incubation and onset of symptoms from case to case.

If these data issues can be dealt with at least partially, the research challenge in using human incidence data is to understand the second- or third-order connections between the soil fungus and reported cases of the disease. There are essentially two hypothesized parts to the role of climate (Kolivras and Comrie 2003) that need to be evaluated. First, existing *Coccidioides* spores present in dry soil need increased soil moisture (via precipitation) to grow the fungus, followed by a dry period during which fungal hyphae desiccate and form spores. Second, wind or other disturbance is required to disperse the spores for inhalation by a host. The relative roles of these climate factors in the seasonality and outbreaks of coccidioidomycosis are not clearly understood. My principal goals in this article are therefore to analyze the postulated climate and dust relationships to fungal growth and dispersion and evaluate their respective roles.

Two subquestions are also considered. First, southern Arizona has a bimodal annual precipitation pattern with one peak in summer and one in winter (Sheppard et al. 2002), but county-level coccidioidomycosis reports in the past have not clearly reflected an associated bimodal coccidioidomycosis pattern (Kolivras and Comrie 2003). Yet early work and a study using student health service data have noted such a pattern (Hugenholtz 1957; Kerrick et al. 1985). Thus, in this article I examine whether recent county-level reports can shed light on the existence of a bimodal incidence pattern in reported data. Second, in evaluating climatic controls on coccidioidomycosis, the critical date is the date of exposure (spore inhalation) rather than the case report date. A method is required that incorporates this lag as well as the changes in coccidioidomycosis reporting characteristics over time. This article presents such an adaptive data-oriented method for estimating date of exposure.

## Materials and Methods

Tucson and the surrounding areas of Pima County in Arizona are highly endemic for coccidioidomycosis (Kolivras et al. 2001). Pima County coccidioidomycosis case data were obtained from the Arizona Department of Health Services (Phoenix, Arizona) for the period 1992–2003. Reporting was voluntary at the beginning of this period (Ampel et al. 1998), although the data continuity and quality are good relative to previous decades (Kolivras and Comrie 2003). The disease became nationally notifiable in 1995 and reporting by laboratories became mandatory at the state level in 1997 (Komatsu et al. 2003). Although the number of reported cases initially appeared to increase as a result, this effect appears to have been minor because incidence continued to grow in an ongoing epidemic (Komatsu et al. 2003).

Pima County annual mid-year population data were obtained from the U.S. Census Bureau (2004). Environmental data were obtained for the greater Tucson urban area, which contains > 90% of the county population. Both precipitation and dust are good potential predictors of coccidioidomycosis (Kolivras and Comrie 2003; Komatsu et al. 2003). Monthly precipitation data for all five available sites in the Tucson area were obtained from the Western Regional Climate Center (2004) for 1988–2003. In conjunction with the incidence data, the precipitation data enable evaluation of hypothesized soil-moisture–fungal-growth relationships. Ambient concentrations of atmospheric particulate matter with a diameter < 10 μm (PM_10_) were obtained from the Pima County Department of Environmental Quality (2004) for the five stations with data from 1991–2003. The PM_10_ data are a direct measure of airborne dust, and because this size threshold includes the typical spore size, these data should be proportionally related to the hypothesized windblown spore concentrations. Precipitation and PM_10_ values were averaged across sites to provide a single time series of the areawide mean for each.

With regard to analyzing the hypothesized climatic controls on coccidioidomycosis, the most relevant information to extract from the incidence data is the date that each patient most likely inhaled the fungal spore (i.e., exposure date). The coccidioidomycosis incidence data include three possibly useful dates to approximate exposure date: estimated date of onset of symptoms (“onset date”), diagnosis date, and report date (although many cases do not have all three dates recorded). Onset date is potentially the most useful of the three, but it is only available for about one-third of the cases, and that proportion varies considerably over time. Ideally, the onset date accounts for some of the variable lag between exposure and reporting; although it is imprecise, it is likely the most accurate index of exposure date. Conversely, the diagnosis date is more precise but the exposure-to-diagnosis lag, which varies from case to case and is longer than the exposure-to-onset lag, has to be estimated in some way. Diagnosis dates are available for most cases. Report dates are, de facto, available for all cases, but they are the most lagged in time from the exposure date; exposure-to-report lags therefore display the greatest variability and are least likely to provide useful links to climate.

Exploration of the various lags and dates indicated no consistent bias or pattern that could be satisfactorily corrected via simple adjustments, such as an overall mean onset-to-diagnosis delay. Instead, the mean onset-to-diagnosis and onset-to-report lag times were calculated for each individual month in the record (rather than averaged across the entire time series). These temporally adaptive empirical lags were smoothed with a 3-month moving average, centered on the middle month, and then used to estimate exposure dates. For cases with an onset date, the exposure date was estimated to be 14 days earlier to allow for the incubation period (Kolivras and Comrie 2003); for cases without an onset date but with a diagnosis date, the exposure date was estimated to occur earlier by the number of days for that month-specific onset-to-diagnosis lag plus 14 days; for cases with only a report date, the exposure date was estimated to occur earlier by the number of days for that month-specific onset-to-report lag plus 14 days. For example, a case reported on 24 November 2003 might have a diagnosis date of 24 July 2003 and no onset date. Based on the mean of other reports with onset dates in November 2003 (actually the October through December 2003 mean), the onset–diagnosis lag is 10 days, so this case would be estimated to have had an onset date of July 14, and thus an estimated exposure 14 days before, on 30 June.

There were 3,283 cases in the data set; 3,181 of these had diagnosis dates, but only 1,089 had onset dates. The proportion of the latter each month and the length of lag for either varied inconsistently over time, necessitating this set of temporally adaptive adjustments. Onset–diagnosis lags had a mean of 12.6, a median of 11.5, a standard deviation of 5.9, a minimum of 2, and a maximum of 32 days; onset–report lags had respective values of 43.0, 44.0, 19.1, 8, and 99 days. Monthly case totals based on estimated exposure were computed and converted to incidence rates per 100,000 of population using linearly interpolated monthly population estimates.

To analyze the lagged relationships and the relative climatologic significance of different times of year, the data were grouped into seasons. Seasonal analyses are advantageous for several reasons: *a*) they are a useful way of dividing the year into alternating wet and dry periods, *b*) they facilitate identification of recurring times of the year that are important, *c*) seasonal aggregation avoids the monthly variability that characterizes the region and leads to overly complex analyses, and *d*) it is analytically and conceptually simpler to compute and understand seasonal lag relationships. In the southwestern United States, seasons are defined principally by precipitation rather than the thermally based spring, summer, fall, and winter sequence typical of middle-latitude locations (Sheppard et al. 2002). Seasonal groupings are widely used for similar kinds of climate analyses (Crimmins and Comrie 2004). Seasons were defined by monthly sequences that captured the predominant seasonal maxima and minima for each variable.

Stepwise regression of the 1992–2003 seasonal data was used to model coccidioidomycosis rates from concurrent PM_10_ (hypothetically related to spore dispersion and therefore exposure) and concurrent and lagged antecedent precipitation (hypothetically related to fungal growth). Previous work has shown that the relevant climate conditions may be different for each coccidioidomycosis season (Kolivras and Comrie 2003), and therefore each season was modeled separately. Models were cross-validated on independent data points using a leave-one-out jackknife method. Because coccidioidomycosis reporting before 1997 may not have been consistent, the same modeling analysis was run on a subset of the data covering just the improved reporting period from 1997 through 2003 for confirmatory purposes.

## Results

**Figure 1:**
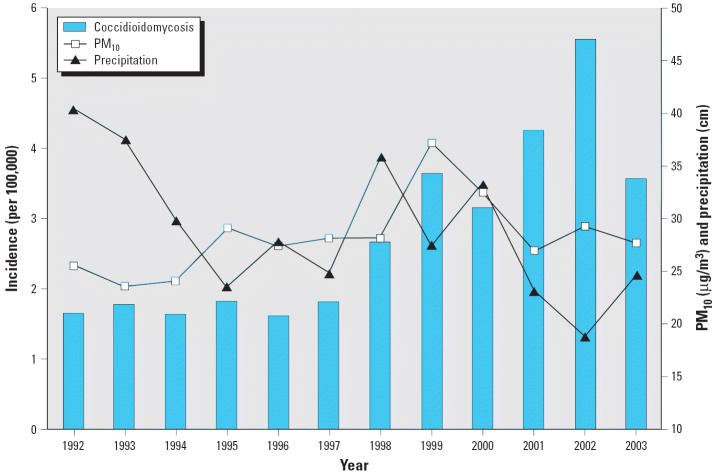
Annual coccidioidomycosis incidence based on estimated exposure date for Pima County, Arizona, with total annual precipitation and mean annual PM_10_ concentrations across sites in the Tucson region.

Application of the estimated exposure date methodology resulted in a time series of coccidioidomycosis incidence, as defined above. An annual plot shows the epidemic in recent years, which coincides with an ongoing regional drought as well as variability in PM_10_ (Figure 1). The 2003 decrease may end up being less pronounced after some reports recorded later in 2004 (unavailable at the time these study data were acquired) are estimated to have been 2003 exposures. Analysis of similar data for the Phoenix area attributed the increase in coccidioidomycosis to climate-related factors (Komatsu et al. 2003).

**Figure 2:**
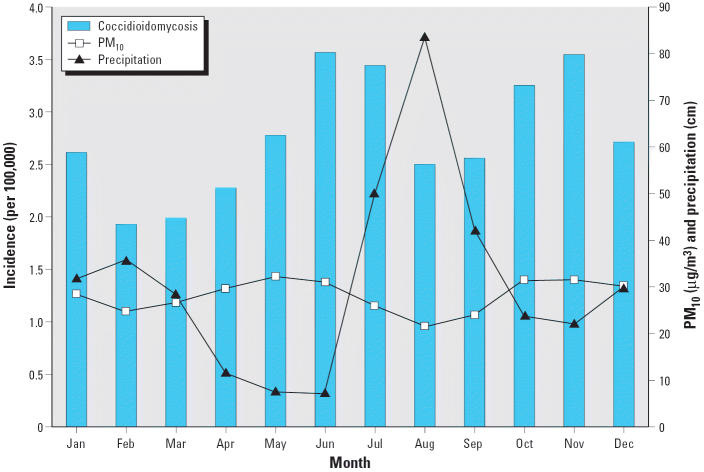
Mean monthly coccidioidomycosis incidence in Pima County, Arizona, based on estimated exposure date, with mean monthly precipitation and mean monthly PM_10_ concentrations, 1992–2003.

Average monthly coccidioidomycosis rates based on estimated exposure dates display obvious seasonal behavior (Figure 2), but with greater clarity than in previous studies. A bimodal pattern with peaks in June–July and October–November is apparent, along with relatively lower incidence in August–September and February–March. PM_10_ concentrations follow an inverse relationship with soil moisture, falling during wet periods and rising during dry periods (Figure 2). Monthly coccidioidomycosis rates are largely consistent with the hypothesis of increased dust exposure leading to increased disease incidence. On the average at least, the less dusty months of the year coincide with lower coccidioidomycosis exposure rates, and elevated rates coincide with or follow the dustier months. Although it is tempting to draw a similar first-order inverse connection between precipitation and incidence at the overall mean monthly level, it is important to recall that this is likely valid for the immediate dust-inhibiting role of rainfall (precipitation has a strong negative correlation with dust) but not likely for its antecedent fungal growth and desiccation role. Thus, although a wet–dry precipitation sequence occurs during the several months before each of the annual coccidioidomycosis peaks on average, closer examination shows that the amount of precipitation and the matching responses as well as the time lags for each are inconsistent. This underlines the importance of investigating the role of antecedent moisture at time scales longer than a season or year.

The monthly averages presented in Figure 2 enabled the definition of seasonal groupings centered on the periods of maxima and minima. Coccidioidomycosis seasons for estimated exposure dates consist of a winter decrease that occurs January through April, a foresummer peak that is seen May through July, a monsoon decrease that takes place in August and September, and a fall peak that is experienced October through December. The same seasons were used for monthly PM_10_ concentrations because they had similar periods of maxima and minima, and because they needed to match the coccidioidomycosis seasons for analysis. For precipitation, the winter peak occurs between December and March, followed by the driest time of the year during the arid foresummer from April through June. The monsoon is the most distinctive aspect of the region’s climate, bringing rainfall during July, August, and September, after which conditions become dryer in a brief fall during October and November (Crimmins and Comrie 2004). Because precipitation is hypothesized to affect fungal growth months or years before the exposure date, it is not necessary to have precipitation seasons exactly match the monthly groupings for the other variables. Thus, for example, it is more meaningful to use July through September for monsoon precipitation and relate that seasonal peak to coccidioidomycosis in subsequent seasons. For simplicity, the names of the seasons are kept the same across all variables.

Adjusted *R*^2^ values for the four seasonal models and standardized (β) coefficients for the variables found to be significant in each model are shown in Table 1. All four models explained significantly high to very high proportions of the variance in coccidioidomycosis rates. It is notable that the strongest relationships do not occur simply in a wet–dry sequence in the season immediately before a rise in coccidioidomycosis rates. A remarkable result is the positive role of precipitation during the arid foresummer for coccidioidomycosis occurring in all subsequent seasons up to 2 years later. One implication is that precipitation during this hottest and driest part of the year (April through June), as opposed to other wetter seasons, is most favorable for *Coccidioides* growth in the environment. This is typically a time of soil desiccation and vegetation dormancy, so the ability to grow opportunistically in the foresummer may be a competitive advantage of *Coccidioides* over other soil biota. A second implication is that fungal spores produced after a wet period in the foresummer may accumulate in the soil and remain viable for several years. Consistent with this hypothesis, monsoonal precipitation does not appear in any model within a 3-year lag, and in only one at 4 years.

Ambient dust levels, as an index of potential spore dispersion, are positively associated with concurrent coccidioidomycosis rates in winter and the foresummer. Dust is not a useful predictor of coccidioidomycosis rates during the monsoon or the fall. Yet wetter conditions in fall appear to decrease concurrent coccidioidomycosis rates and in the winter immediately after, presumably via dispersion inhibition due to greater soil moisture.

The analysis was repeated on the more reliable 1997–2003 data period to check for consistency. This step reduced the modeled *n* from 12 to 7, which decreased statistical reliability, and therefore detailed results are not shown. Nonetheless, although the full set of significant variables differed for each model, the results from the shorter period showed some similarities with the longer period. Those variables that were significant in both the full-period and the later-period models are noted by asterisks in Table 1. Both sets of models have in common the foresummer precipitation 1 or 2 years before the predicted coccidioidomycosis season, as well as concurrent fall precipitation for fall coccidioidomycosis incidence.

**Figure 3:**
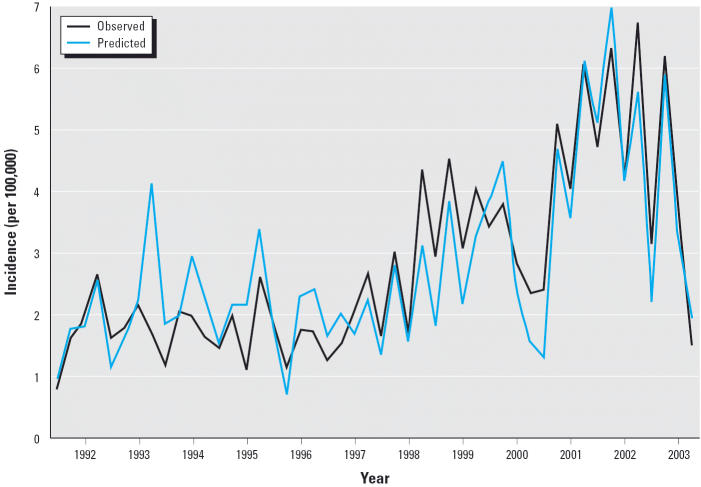
Observed coccidioidomycosis incidence in Pima County, Arizona, and predicted incidence from the cross-validated seasonal models, based on estimated exposure date.

The overall time series of observed and predicted seasonal coccidioidomycosis incidence (for the full period) is shown in Figure 3. The combined predictions of all four multivariate seasonal models are in close agreement with observations, with an overall cross-validated *R*^2^ of 0.80, and a cross-validated mean absolute error of 0.53 cases per 100,000, or about 19% of the mean incidence. The proportions of model-oriented (systematic) error versus data-oriented (unsystematic) error were 14 and 86%, respectively (Comrie 1997), implying that the model is well specified and that noisy data are responsible for most of the error. To further isolate the role of the foresummer, antecedent foresummer precipitation alone was regressed on coccidioidomycosis incidence in fall, winter, foresummer, and the monsoon in the relevant period 1.5–2 years later. The resulting cross-validated *R*^2^ between observations and combined predictions of all four antecedent foresummer-based models was 0.27.

## Discussion

The development of a method to estimate *Coccidioides* spore exposure date from coccidioidomycosis incidence data has enabled the production of a relatively homogeneous time series. This approach reveals a strong bimodal seasonality of the disease in Pima County, Arizona, consistent with earlier findings based on other data (Hugenholtz 1957; Kerrick et al. 1985), a pattern that until now was not clearly seen in the regular reported data. On average, peaks in exposure to the fungal spores occur in June–July and in October–November, consistent with the drier and dustier months of the year. Fewer exposures occur in February–March and August–September, consistent with the timing of the wetter and less dusty months.

Multivariate models of the incidence data series indicate that concurrent dispersion conditions are important during fall (via precipitation) and in winter and the arid foresummer (via PM_10_). However, the most striking result of this study is the dominant role of precipitation during the normally arid foresummer 1.5–2 years before the season of exposure. Even when considered alone, April–June precipitation accounts for more than one-quarter of the overall variance in subsequent seasonal coccidioidomycosis incidence. When other antecedent and concurrent seasonal conditions are included as predictors, the combined seasonal models explain a significant and large proportion of the variance in coccidioidomycosis incidence. The model is relatively simple in structure compared with other studies (Kolivras and Comrie 2003; Komatsu et al. 2003). The model uses only lagged seasonal precipitation and concurrent seasonal dust and precipitation, yet it clearly captures both the seasonality and the trends in the incidence data. The bulk of the error is associated with noise in the data, so future improvements to the model are likely to result from improved data and a longer length of record with a larger model *n*.

An improved understanding of the climatic factors behind outbreaks of coccidioidomycosis will enable better timing of environmental sampling for *Coccidioides* and any related mitigation efforts, separation of environmental factors from population and other factors affecting outbreaks, and the potential for development of an advance warning system before an outbreak. The results of this work provide strong support for the two hypothesized relationships between climate and coccidioidomycosis, namely, fungal growth in the longer term and spore dispersion and exposure in the short term. Furthermore, the relative simplicity and strength of these results relative to earlier studies (Kolivras and Comrie 2003; Komatsu et al. 2003) lend considerable confidence to the potential for the development of an operational disease forecast model. The ability to define a critical event, such as precipitation during the foresummer, might enable mitigation procedures immediately after the event as well as provide a useful public health tool with an 18-month lead time on expected incidence of coccidioidomycosis. Future work will need to evaluate how specific these findings are to southern Arizona versus other regions in which *C. posadasii* is also endemic, and whether similar relationships also apply to *C. immitis* in California. It will also be valuable to test how a more complex model (Komatsu et al. 2003) and this simpler model compare against data from other locations and over time.

## Figures and Tables

**Table 1:** Model performance and standardized (β) coefficients for the four seasonal regression models predicting coccidioidomycosis rates from concurrent PM_10_ and antecedent precipitation, 1992–2003 (significance in parentheses).

Measure	Foresummer	Monsoon	Fall	Winter
Performance				
Adjusted *R*^2^	0.98 (≤ 0.001)	0.60 (0.006)	0.61 (0.006)	0.95 (≤ 0.001)
Cross-validated *R*^2^	0.95 (≤ 0.001)	0.66 (0.001)	0.66 (0.001)	0.74 (≤ 0.001)
Dust				
PM_10_	0.75 (≤ 0.001)			0.44 (≤ 0.001)
Precipitation*^a^*				
Winter-0	N/A*^b^*	N/A	N/A	
Fall-0	N/A	N/A	−0.49* (0.029)	−0.36 (0.004)
Monsoon-0	N/A			
Foresummer-0	0.47 (≤ 0.001)			0.49 (≤ 0.001)
Winter-1	0.20 (0.023)			−0.33 (0.004)
Fall-1	−0.26 (0.030)			
Monsoon-1				
Foresummer-1		0.45 (0.044)	0.73* (0.004)	0.56* (≤ 0.001)
Winter-2				
Fall-2				
Monsoon-2				
Foresummer-2	1.36* (≤ 0.001)	0.64* (0.008)		
Winter-3				
Fall-3				
Monsoon-3				
Foresummer-3				
Winter-4				
Fall-4				N/A
Monsoon-4	−0.93 (≤ 0.001)		N/A	N/A
Foresummer-4		N/A	N/A	N/A

a For precipitation variables, Fall-0 denotes the concurrent fall, Winter-4 denotes the winter occurring 4 years earlier, and so on, ordered from most to least recent.

b Seasons falling before or after the period including the concurrent season through 4 years earlier are marked as not applicable (N/A).

* Model variables that were also present in a 1997–2003 subset analysis, signifying those variables that were significant in both the full-period and the later-period models.

## References

[b1-ehp0113-000688] Ampel NM, Mosley DG, England B, Vertz PD, Komatsu K, Hajjeh RA (1998). Coccidioidomycosis in Arizona: increase in incidence from 1990 to 1995. Clin Infect Dis.

[b2-ehp0113-000688] CDC2004a. MMWR Morbidity Tables—Table II (Part 1): Provisional Cases of Selected Notifiable Diseases, United States, Week Ending October 23, 2004. Atlanta, GA:Centers for Disease Control and Prevention. Available: http://wonder.cdc.gov/mmwr/mmwr_reps.asp?mmwr_year=2004&mmwr_week=42&mmwr_table=2A [accessed 2 November 2004].

[b3-ehp0113-000688] CDC (2004b). West Nile virus activity—United States, September 15–21, 2004. MMWR Morb Mortal Wkly Rep.

[b4-ehp0113-000688] Comrie AC (1997). Comparing neural networks and regression models for ozone forecasting. J Air Waste Management Assoc.

[b5-ehp0113-000688] Crimmins MA, Comrie AC (2004). Interactions between antecedent climate and wildfire variability across southeast Arizona. Int J Wildland Fire.

[b6-ehp0113-000688] Fisher MC, Koenig GL, White TJ, Taylor JW (2002). Molecular and phenotypic description of *Coccidioides posadasii* sp. nov., previously recognized as the non-California population of *Coccidioides immitis*. Mycologia.

[b7-ehp0113-000688] Hugenholtz P1957. Climate and coccidioidomycosis. In: Proceedings of the Symposium on Coccidioidomycosis, Phoenix, Arizona. Publication 575. Washington, DC:U.S Public Health Services, 136–143.

[b8-ehp0113-000688] Jinadu BA1995. Valley Fever Task Force Report on the Control of *Coccidioides immitis*, Kern County. Bakersfield, CA:Kern County Health Department.

[b9-ehp0113-000688] Kerrick SS, Lundergan LL, Galgiani JN (1985). Coccidioidomycosis at a university health service. Am Rev Respir Dis.

[b10-ehp0113-000688] Kolivras KN, Comrie AC (2003). Modeling valley fever incidence based on climate conditions in Pima County, Arizona. Int J Biometeorol.

[b11-ehp0113-000688] Kolivras KN, Johnson P, Comrie AC, Yool SR (2001). Environmental variability and coccidioidomycosis (valley fever). Aerobiologia.

[b12-ehp0113-000688] Komatsu K, Vaz V, McRill C, Colman T, Comrie A, Sigel K (2003). Increase in coccidioidomycosis—Arizona, 1998–2001. MMWR Morb Mortal Wkly Rep.

[b13-ehp0113-000688] Maddy K (1965). Observations on *Coccidioides immitis* found growing naturally in soil. Ariz Med.

[b14-ehp0113-000688] National Research Council2001. Under the Weather: Climate, Ecosystems and Infectious Disease. Washington, DC:National Academy Press. 25057537

[b15-ehp0113-000688] Pappagianis D1988. Epidemiology of coccidioidomycosis. In: Current Topics in Medical Mycology, Vol 2 (McGinnis MR, ed). New York:Springer-Verlag, 199–238. 10.1007/978-1-4612-3730-3_6 3288356

[b16-ehp0113-000688] Park BJ Sigel K Vaz V Komatsu K McRill C Phelan M2005. An epidemic of coccidioidomycosis in Arizona associated with climate changes, 1998–2001. J Infect Dis. Available: http://www.journals.uchicago.edu/JID/journal/rapid.html (online 20 April 2005). 10.1086/430092 15871133

[b17-ehp0113-000688] Pima County Department of Environmental Quality2004. Daily Particulate Matter Monitoring Data. Tucson, AZ:Pima County Department of Environmental Quality.

[b18-ehp0113-000688] Sheppard PR, Comrie AC, Packin GD, Angersbach K, Hughes MK (2002). The climate of the U.S. Southwest. Clim Res.

[b19-ehp0113-000688] U.S. Census Bureau2004. County Population Estimates. Washington, DC:United States Census Bureau. Available: http://www.census.gov/popest/archives/1990s/co-99-08/99C8_04.txt [accessed 2 November 2004].

[b20-ehp0113-000688] Western Regional Climate Center2004. Arizona Climate Summaries. Reno, NV:Western Regional Climate Center. Available: http://www.wrcc.dri.edu/summary/climsmaz.html [accessed 2 November 2004]

